# A194 PATTERNS OF INFLAMMATORY BOWEL DISEASES IN NORTHWESTERN ONTARIO

**DOI:** 10.1093/jcag/gwae059.194

**Published:** 2025-02-10

**Authors:** C Lee, A Farahvash, S Smith, M Clark, A Zezos, R Siddiqui, S Dubois, C Rumbolt, J Green, P Zezos

**Affiliations:** NOSM University - Thunder Bay Campus, Thunder Bay, ON, Canada; NOSM University - Thunder Bay Campus, Thunder Bay, ON, Canada; NOSM University - Thunder Bay Campus, Thunder Bay, ON, Canada; NOSM University - Thunder Bay Campus, Thunder Bay, ON, Canada; NOSM University - Thunder Bay Campus, Thunder Bay, ON, Canada; NOSM University - Thunder Bay Campus, Thunder Bay, ON, Canada; NOSM University - Thunder Bay Campus, Thunder Bay, ON, Canada; NOSM University - Thunder Bay Campus, Thunder Bay, ON, Canada; NOSM University - Thunder Bay Campus, Thunder Bay, ON, Canada; NOSM University - Thunder Bay Campus, Thunder Bay, ON, Canada

## Abstract

**Background:**

Geographic disparity adds to the challenges of caring for patients with inflammatory bowel disease (IBD) in Canada. Northwestern Ontario is one of the least densely populated regions among Canadian provinces, with limited existing research examining patients affected by IBD.

**Aims:**

We sought to 1) describe the clinical trends and characteristics of patients with IBD in Northwestern Ontario and 2) compare the course of disease among patients residing in urban versus rural areas.

**Methods:**

Thunder Bay is the major site for specialized care in Northwestern Ontario, allowing for the inclusion of approximately all IBD patients in the region. This retrospective study involved data collected over a 25-year period from January 1, 1999 to October 1, 2024. Northwestern Ontarians with diagnostic confirmation of IBD were included. Deceased patients were excluded. Participants were identified using billing codes and keywords from electronic records of three gastroenterologists practicing at the Thunder Bay Regional Health Sciences Centre, the only tertiary care centre in Northwestern Ontario. Data were collected from clinic and hospital records. Student’s t-test and chi-squared test were used in statistical analysis.

**Results:**

Among 2115 patients who were screened, this preliminary analysis identified 1061 IBD patients, including 546 (51.5%) with ulcerative colitis, 481 (45.3%) with Crohn’s disease, and 34 (3.2%) with unclassified colitis. Mean age was 52.6±17.3 years and 49.9% were female. Mean age at diagnosis was 36.2±17.0 years and median time to diagnosis was 5 months. There were significantly more smokers among patients with Crohn’s. Based on the last clinical encounter, 692 (65.2%) patients were in clinical remission, 174 (16.4%) were in active disease, and 186 (17.5%) were lost to follow up. Current medications included biologics (369, 34.8%), 5-ASA (366, 34.5%), immunomodulators (119, 11.2%), and glucocorticoids (71, 6.7%). Throughout the course of disease, mean number of Emergency Department visits and hospital admissions were 2.6±4.7 and 1.2±2.4 respectively, and 325 (30.6%) patients underwent IBD-related surgery.

The cohort included 752 (70.9%) urban residents and 309 (29.1%) rural residents. No significant difference was observed between urban and rural residents, including age, sex, disease type, prevalence of biologic use, disease severity based on the Montreal classification, or current disease status.

**Conclusions:**

This study characterizes the patient population and clinical trends of IBD in a low population density region in Ontario. There appears to be homogeneity between rural and urban residents, with comparable patient and disease features, and access to medications including biologics.

**Funding Agencies:**

None

